# A national prevalence and profile of single and multiple developmental delays among children aged from 1 year up to 12 years: an Egyptian community-based study

**DOI:** 10.1186/s13034-022-00498-3

**Published:** 2022-08-05

**Authors:** Ammal M. Metwally, Ali M. Abdallah, Ebtissam M. Salah El-Din, Zeinab Khadr, Ehab R. Abdel Raouf, Nahed A. Elghareeb, Rehan M. Saleh, Manal H. Abuelela, Hala A. Amer, Hasanin M. Hasanin, Mohamed A. Abdel Mawla, Sara F. Sallam, Inas R. El-Alameey, Samia M. Sami, Ghada A. Abdel-Latif, Mohamed Abdelrahman, Manal A. Shehata

**Affiliations:** 1 Community Medicine Research Department/ Medical Research and Clinical Studies Institute, National Research Centre (Affiliation ID: 60014618), Dokki, Cairo, Egypt; 2 Quantitative Methods Department, Aswan University, Giza, Egypt; 3 Child Health Department/ Medical Research and Clinical Studies Institute, National Research Centre (Affiliation ID: 60014618), Dokki, Cairo, Egypt; 4 Department of Statistics, Faculty of Economics and Political Science, Cairo University, Giza, Egypt; 5 The Social Research Center of the American University in Cairo, Cairo, Egypt; 6 Department of Child with Special Needs/Medical Research and Clinical Studies Institute, National Research Centre (Affiliation ID: 60014618), Dokki, Cairo, Egypt; 7 Prevention of Disability General Directorate, Ministry of Health and Population, Giza, Egypt; 8 Public Health Department, Research Institute of Ophthalmology, Giza, Egypt; 9 Pediatrics Dept., Medical Research and Clinical Studies Institute, National Research Centre (Affiliation ID: 60014618), Dokki, Cairo, Egypt; 10 Department of Infection Control, King Saud Medical City, Riyadh, Saudi Arabia; 11 Faculty of Applied Medical Sciences, Clinical Nutrition Department, Taibah University, Riyadh, Saudi Arabia; 12 Public Health and Community Medicine, Medical Research and Clinical Studies Institute, National Research Centre (ID: 60014618), P.O. 12622, Dokki, Giza, Egypt

**Keywords:** Developmental delays, Multiple delays, Risk and protective factors

## Abstract

**Objective:**

This study aimed at providing a national prevalence of single and multiple developmental delays (DDs) among 41,640 Egyptian children aged 1 to 12 years and exploring DDs’ associated risk and protective factors.

**Methods:**

A national household survey from eight governorates of Egypt representing the four major subdivisions of Egypt was conducted through systematic probability proportionate to size. All enrolled children were assessed according to Vineland Adaptive Behavior Scales, (VABS) as a reliable screening questionnaire for identifying categories of DDs that were verified by pediatrics’ specialists.

**Results:**

The overall prevalence of children with DDs was 6.7%. The prevalence of a single DD was 3.9% versus 2.8% multiple DDs. Communication deficit was the most prevalent type (5.3%). Lower prevalence was identified for fine motor delay (1.0%), gross motor delay, and socialization deficit (1.5% each). Whereas deficits in daily life skills (self-help and adaptive behavior delay) amounted to 2.3%. Living without mothers and/or fathers in homes was associated with increased odds of having DDs by one and a half times (OR = 1.72 and OR = 1.34 respectively). Multiple logistic regression analysis revealed the most predictors for DDs including children who suffer from convulsions after birth (OR = 3.10), low birth weight babies (OR = 1.94), male sex (OR = 1.75), mothers having health problems during pregnancy (OR = 1.70) and belonging to middle socioeconomic status (OR = 1.41). Children who suffered from cyanosis after birth was found to be at risk for any or multiple DDs. Difficult labor was significantly associated with increased odds for multiple DDs (OR = 1.55). Higher paternal and maternal education was associated with decreased odds to have any DDs by 40% (OR = 0.60 and OR = 0.58 respectively).

**Conclusions:**

The detected prevalence of DDs is within the estimated range of prevalence of DDs for the pediatric population. The majority of the detected risk factors are preventable. Developmental screening is recommended to be implemented in all primary care settings as a routine practice.

**Supplementary Information:**

The online version contains supplementary material available at 10.1186/s13034-022-00498-3.

## Background

Developmental delay (DD) indicates a broad spectrum of impairments of developmental milestones that are proper to a child’s age. It is defined under main streams or domains including the motor, adaptive abilities, cognitive, language, and social-emotional domains [1]. Overall, DDs are prevalent and can include either one domain or multiple domains [2].

Community-based studies in America, Europe, and Asia reported the prevalence of childhood DDs varying from 4 to 17% [3–5]. While, in Egypt, data regarding the developmental problems in children are scarce. Estimates from small-scaled studies showed a prevalence of 3% to 9% [6, 7].

Globally, developmental delays cause more morbidity over a lifetime than any other chronic case [8]. Several works of literature have addressed the negative consequences of childhood DDs, including emotional, behavioral, and health troubles later in life [9], affected the parent–child relationship and other familial and social problems [10], academic problems, and delayed educational achievement [11], and the economic influences on the families and society [12].

The etiology for most of DDs is idiopathic. There is no exact underlying cause that can be determined, but rather, risk factors can contribute to the problem. These factors have been categorized into environmental, psychosocial, and biological groups of factors, which are often act in synergy [13, 14]. Identifying these risk factors and which factors can provide protection and resilience in children, may be necessary to control the problem [15].

Provision of Care and Support is recommended for a child who experienced any sort of delay or disability. In absence of screening, vulnerable children with developmental delay will be missed from being early detected, receiving their appropriate interventions, and being subjected to developmental disabilities. Early detection of developmental delays and proper intervention can definitely change the child’s developmental trajectory [2]. A crucial initial stage for early intervention plans is estimating the prevalence of developmental delay and identifying the types of delays. In Egypt, not much data is available about types of DDs and their associated risk factors through using a reliable screening questionnaire.

## Methods

### The aim

This survey aimed at providing a national estimate for the occurrence of types of developmental delays, single and multiple delays at a community level by using a reliable screening questionnaire and exploring DDs’ associated risk and protective factors out of the studied epidemiological, sociodemographic, prenatal, neonatal, and postnatal factors.

### Study type, design, and setting

A Cross-sectional national community-based prevalence survey was conducted in 8 Governorates representing all geographic regions of Egypt according to their population density. The whole study was conducted along 24 months

### Target group

The study targeted parents or caregivers of all children aged 12 months up to 12 years at the visited houses.

### Inclusion and exclusion criteria

Any child in the age range 12 months to 12 years who experienced normal milestones for his age as well as any child who met the definition of developmental delay was included in the current study whether previously identified as having DDs or newly diagnosed during the current study. A developmental delay (DD) is defined as slow to reach milestones in one or more of the areas of development (Gross or fine motor, communication (Language/cognition), socialization, or adaptive or daily life skills) in an expected way for a child's age [1]. The authors of this study focused on early identification of children whose development may be delayed to prevent subsequent disability. Consequently, children with known or previously diagnosed developmental disabilities were not included in the estimated prevalence. Definition of developmental disability includes impairment of a body structure or function which occurs during developmental period accompanied with limitation of activities and restriction of participation [16]. This means that all children who have a diagnosed developmental disability were not eligible for this study. The excluded children were those with diagnosed genetic disorders (e.g., Down syndrome, Turner syndrome, or Fragile-X syndrome) or with disabilities affecting vision, hearing, movement, thinking, learning, or social relationships. This group of children was referred by the investigators to specialized centers belonging to Ministry of Health and population (MOHP) for management and rehabilitation.

### Sampling frame and cluster preparation

Three sampling frames were chosen as three stages; The first sampling frame used was the comprehensive list of the governorates in Egypt according to the enumeration census from the Central Agency for Public Mobilization and Statistics (CAPMS) [17]. The number and percent distribution of the population according to governorates in 2017 census revealed that population percentage in urban governorates reached 17.1% of the total population, 43% in Lower Egypt versus 38% in Upper Egypt, while Frontier governorates only represented 1.7% of the total population in the same year. In the first stage, a representative sample of 8 governorates was selected to represent the main geographic areas in Egypt including *One urban (Cairo)*, *3 Upper Egypt (Fayoum, Assuit, and Aswan), 3 Lower Egypt (Damietta, Dakahlia, and Gharbia)* and *One border -Frontier- (Marsa Matrouh*).

In the second stage, a representative sample of cities and local units were selected from each governorate. In this selection process, the design of the sample took into consideration the differences in human development within each governorate. Using the human development index produced by the Economic Research Forum and CAPMS in Egypt [18], each governorate was divided into three categories according to their human development scores, namely low, middle, and high social class. One city for urban areas and a local unit for rural areas were selected from each category for each governorate. In the third stage, for urban areas, the selected cities will be divided into city blocks then choosing one or two blocks for surveying. For the rural areas, one or two villages were selected from the selected local units. A total of 45-blocks ensured both the adequate sample size and heterogenicity of data collected. In this stage, households in the selected city and villages blocks were screened. The sample was allocated to be proportional to the size of large governorates. While governorates with relatively small populations were assigned random sample sizes with weights adjusted during data analysis.

### Survey sample size

Sample size calculation is based on the estimated prevalence of each individual domain of developmental delays with Sample size calculation is based on the estimated prevalence of 3% in Barbados to 67% in Chad with developmental delays in 63 low- and middle-income surveyed countries [19]. The least prevalence was taken into consideration to ensure the largest sample size at a level of accuracy set at 0.0049 (margin of error), confidence limit 95%, reliability of the questionnaires used for detecting DDs was 80%. Accordingly, targeting 40,000 children is expected to ensure the provision of accurate and representative national estimatesof DDs. This number was expected to be targeted from 22,026 surveyed houses along 45 blocks. The large representative sample in the screening phase aims at ensuring data accuracy.

### Study instruments

Through face-to-face interviews with the parents or caregivers, three parts of a household-developed questionnaire were fulfilled.

*The first part* of the household questionnaire collected information on housing and sociodemographic characteristics including age, sex, birth order, number of siblings, maternal age, residence, and parental education and occupation [18].

*The second part* of the questionnaire collected information about the epidemiological, prenatal, neonatal, and postnatal child and maternal medical histories.

*The third part* was a developmental screening for detecting any developmental delay in one or more domains among children aged 12 months to 12 years. In Egypt, as that in most of the developing countries, developmental monitoring of young children is not an integral part of health care services. Owing to cost, time, and lack of professional specialists, the research team depended on developmental screening. Developmental screening is an approach in which groups of children are screened to ascertain whether they have developmental delay, by testers who do not necessarily have a continuous relationship with the families or access to health or social information other than that provided by the screening instrument [20].

The Arabic version of Vineland Adaptive Behavior Scales, (VABSA) [21] was used in this study for assessment of developmental functions. The Arabic version was validated with good reliability and validity and used in several studies in Arabic Countries [21]. Adaptive behavior is the performing of everyday activities needed to function, meet environmental requirements, care for oneself, and interact with others efficiently and independently [22]. Adaptive behavior describes essential characteristics of functioning for all persons and may constitute the fundamental developmental outcome in psychology [23].

- *The Vineland Adaptive Behavior Scales (VABS)* is one of the most commonly used measures of parent-reported adaptive behavior for individuals from birth through adulthood [24]. It is a diagnostic tool that measures adaptive functioning across four domains: Communication, Daily Living Skills, Socialization and Motor Skills. It supports diagnosis of intellectual and developmental disabilities, autism, and developmental delay [25]. VABS provides specific information useful in exploration of individual strengths and weaknesses. The scales are used in a variety of clinical, educational, and research settings [26].
- The measured four domains integrated eleven subdomains. In the Communication Domain, subdomains of receptive, expressive, and written language are measured. The Daily Living Skills Domain includes subdomains of personal, domestic and community skills. The Socialization Domain addresses interpersonal relationships, play and leisure time, and coping skills subdomains. The Motor Skills domain measures both gross and fine motor skill subdomains. Each subdomain consists of multiple items. Researchers of this study preferred to assess gross and fine motor skills independently because they may potentially differ in their developmental trajectories, affording different types of interactions which may predict later developmental outcomes.
- The Vineland has two versions, one that can be completed by parents or caregivers independently, and one that is administered to parents or caregivers via a trained interviewer. The Vineland survey form, which is administered by a trained interviewer, was used in this study. All items are scored on a 3-point scale of behavior frequency, with 0 indicating never, 1 indicating sometimes or partially, and 2 indicating usually. The starting point is the item keyed in the record booklet to the individual's chronological age. After completing the interview, the interviewer completes a score summary page for recording and profiling derived scores. The mean of the adaptive behavior composite score is 100 with SD of 15. A delay in specific domain is considered if the score of that domain is 2 SD below the mean (< 70). In this study, the child was classified as having delay or not according to the cutoff of 70.
- The VABS demonstrated good reliability and validity [22]. Specifically, for the survey form, Sparrow et al. reported split-half reliability coefficients ranging from 0.83 to 0.94 across all domains, test–retest reliability coefficients ranging from 0.81 to 0.86, and adequate interrater reliability coefficients equal 0.74. Concurrent validity with the Vineland Social Maturity Scale is adequate with r = 0.55.

Discovered children with low scores on VABS signifying developmental delay were referred to the pediatrician in the health centers of the Ministry of Health and Population (MOHP) as a second phase of the study. Surveyed children have amounted to 41,640. The children who compiled with referral to the MOHP facilities have amounted to 3193. With 2.5% losses and 10.5% negative diagnosis, children who ascertained the diagnosis of developmental delay have amounted to 2778 representing 87% of those who compiled referral.

Data collection was implemented by professional field surveyors. Before the survey implementation, a preparation phase was done to ensure quality control, contacting local authorities. Condensed training sessions about how to conduct the questionnaire in a standardized way were done for 64 social workers (average 6/governorate). The survey was conducted under the supervision of a collaborative team from Cairo Demographic Center (CDC) with the professional team members from the National Research Centre of Egypt (NRC). A pilot study was performed on 80 participants (10 / governorate) to ensure the validity of the questionnaire items through revising and modifying difficulty understood items or language and then re-introduced.

### Statistical analysis

Data were analyzed using Statistical Package for the Social Sciences (SPSS) version 22.0 software (IBM SPSS Statistics for Windows, Version 22.0. Armonk, NY: IBM Corp.). All data were represented by percentages and comparisons between groups were done using odds ratios (OR) and 95% confidence intervals (CI) were calculated in comparison between DDs and children without delays. Probability values (p) < 0.05 were regarded as statistically significant. Logistic regression analysis was done to assess the contribution of each independent variable to predict the odds of developing DDs and multiple DDs based on the values of the independent variables (risk factors for DDs) [27]. All continuous variables were transformed into grouped categorical variables. A significant association is considered if the 95% CI does not include the value 1.0, and a cutoff *p*-value of less than 0.05 is used for all tests of statistical significance in this study.

## Results

The total number of surveyed children was 41,640. Boys represented 51.5% of the whole sample vs. 48.5% for girls. Regarding the age distribution, children aged 6–12 years represented the largest portion 49%. While the presence of neonatal jaundice was the most prevalent perinatal problem with 26.5%. Study population characteristics were shown in Table 1

**Table 1:** Characteristics of the study population

Variable	Total surveyed children N = 41,640 N (%)
*Age*	
1- < 3 years	8382 (20%)
3- < 6 years	12,933 (31%)
6–12 years	20,324 (49%)
*Gender*	
Boys	21,437 (51.5%)
Girls	20,203 (48.5%)
*Perinatal problems*	
Premature children (< 37 weeks gestation)	413 (1%)
Low birth weight (< 2500 mg)	1848 (4.4%)
children suffer from jaundice after birth	11,028 (26.5%)
children suffer from bluish discoloration after birth (Cyanosis)	546 (1.3%)
children suffer from any convulsions	675 (1.6%)
children kept in an incubator for more than two days	3078 (7.4%)
Mothers have any health problem during pregnancy*	2770 (6.6%)
difficult labor**	6092 (14.6%)

*Mothers have any pregnancy complications as iron deficiency anemia, gestational diabetes, hypertension, infection, anxiety, or depression [28]

**Difficult labor refers to prolongation in the duration of labor, typically in the first stage of labor. It can be an important contributor to maternal and perinatal mortality and morbidity if it remains unrecognized or untreated [29]

The overall prevalence of developmental delays was 6.7%. Children aged 3 – < 6 years were the most likely to be diagnosed with any developmental delay with the highest prevalence of 7.9%. The odds of any DDs was significantly twice that of the age group 1- < 3 years (OR = 1.96, CI: 1.73–2.22) but slightly more than children aged 6–12 years (OR = 1.14*, CI: 1.05–1.24). Boys were nearly one and three quarters more likely than girls to be diagnosed with any developmental delays (OR = 1.75, CI: 1.61–1.89).

Children belonging to either the urban or middle class carry almost significantly one and a half the odds for developmental delays than children belonging to either rural or the low or the high class (OR = 1.34, 1.43&1.38 respectively).

The odds for the presence of at least one developmental delay in frontier, upper and lower Egypt governorates was less than the odds in cities by (57%, 52% & 40% respectively) at a significance level less than 5%. Data concerning sociodemographic characteristics are demonstrated in Table 2

**Table 2:** Sociodemographic and Epidemiological characteristics of the surveyed children regarding Developmental delays

Socio-demographic parameters	Surveyed children N = 41,640 N (%)	Healthy children N = 38,862 (93.3%)	Children with any DDs n = 2778 (6.7%)	OR (CI)
*Locality (Urban/Rural)*				
Urban	19,422 (46.6%)	17,935	1291	Urban Vs. Rural 1.34 (1.24–1.45)**
Row %		92.3%	5.8%	
Rural	22,218 (53.4%)	20,927	1487	
Row %		94.2%	7.7%	
*Social class*				
Low	13,586 (32.6%)	12,793	793	Middle vs. low 1.43 (1.30–1.57)**
Row %		94.2%	5.8%	
Middle	13,887 (33.4%)	12,756	1131	Middle vs. high 1.38 (1.26–1.52)**
Row %		91.9%	8.1%	
High	14,167 (34.0%)	13,311	856	
Row %		94%	6%	
*Geographical Distribution*				
Cities	6919 (16.6%)	6184	735	Lower vs Cities 0.596 (0.54-0.66)**
Row %		89.4%	10.6%	
Lower Egypt	15,892 (38.2%)	14,840	1052	
Row %		93.4%	6.6%	
Upper Egypt	14,344 (34.5%)	13,570	774	Upper vs cities
Row %		94.6%	5.4%	
Frontier	4485 (10.8%)	4268	217	Frontiers vs cities 0.43 (0.37-0.50)**
Row %		95.2%	4.8%	
*Sex*				
Male	21,437 (51.5%)	19,655	1782	Males/Females 1.75 (1.61–1.89)**
Row %		91.7%	8.3%	
Female	20,202 (48.5%)	19,207	996	
Row %		95.1%	4.9%	
*Age category*				
1- < 3 years	8383 (20.1%)	8033	349	3- < 6 years vs. 1- < 3 1.96 **(1.73–2.22)
Row %		95.8%	4.2%	
3- < 6 years	12,933 (31.1%)	11,917	1016	
Row %		92.1%	7.9%	
6–12 years	20,324 (48.8%)	18,911	1413	6–12 vs. 1- < 3 years 1.72 (1.53–1.94)**
Row %		93%	7%	

* = significant < 0.05, ** = highly significant < 0.01

Maternal and paternal characteristics of the surveyed children concerning DDs are demonstrated in Table 3. Children with mothers or fathers who had higher education were less likely to be diagnosed with any developmental delay with the least odds for the mothers and fathers who had a college or greater education level (OR = 0.57, 95% CI: 0.50–0.64 and OR = 0.50, 95% CI: 0.44–0.57 respectively).

**Table 3:** Maternal and Paternal characteristics of the surveyed children with respect to Developmental delays

Socio-demographic parameters N = 41,640	Surveyed children N (%)	Healthy children N = 38,862	Children with any DDs n = 2778	OR (95% CI)
*Mother age at giving birth*				
Less than 18	1976 (4.7%)	1845	131	> 35 vs. < 18 1.10 (0.88–1.37)
Row %		93.4%	6.6%	
18 to < 35	35,958 (86.4%)	33,590	2368	18- < 35 vs. < 18 0.99 (0.83–1.19)
Row %		93.4%	6.6%	
> 35	3407 (8.2%)	3161	246	
Row %		92.8%	7.2%	
No mother	299 (0.7%)	254	31	no mother vs. mother at home 1.72** (1.18–2.5)
Row %		89.1%	10.9%	
*Current Mothers Age*				
less than 35	36,344 (87.3%)	35,435	2499	= > 35 vs. < 35 1.10 (0.96–1.26)
Row %		93.4%	6.6%	
= > 35	5296 (12.7%)	3161	246	
Row %		92.8%	7.2%	
*Mothers Education*				
1. Illiterate/read and write to Prep	16,046 (38.5%)	14,825	1221	OR 3 vs. 1 0.57 (0.5–0.64)**
Row %		92.4%	7.6%	
2. High School and technical	18,609 (44.7%)	17,381	1228	OR 2 vs. 1 0.86(0.79-0.93)*
Row %		93.4%	6.6%	
3. University or higher	6674 (16%)	6377	297	
Row %		95.5%	4.5%	
*Fathers Education*				
1. Illiterate/ Read and write to Prep	14,666 (35.2%)	13,499	1167	OR 3 vs. 1 0.50 (0.44-0.57) **
Row %		92.0%	8.%	
2. High School and technical	18,390 (44.2%)	17,222	1168	OR 2 vs. 1 0.78 (0.72-0.85)**
Row %		93.6%	6.4%	
3. University or higher	6662 (16.%)	6385	277	
Row %		95.8%	4.2%	
4. No father at home	1922 (4.6%)	1755	166	no Father vs. Father at home 1.34 (1.14–1.58)**
Row %		91.4%	8.6%	
*Mothers’ work*				
1. Work (paid-unpaid-her own)	6014 (14.4%)	5651	363	OR 2 vs 1 1.13 (1.01–1.26)
Row %		94.0%	6.0%	
2. Unemployed	35,315 (84.8%)	32,935	2383	
Row %		93.3%	6.7%	

*Significant < 0.05, **highly significant < 0.01

Age at giving birth or current mothers age or mothers working condition did not show any association with the occurrence of developmental delays. Meanwhile, living without mothers and/or fathers in homes was associated with increased odds of having DDs by more or less one and a half times (OR = 1.72, 95% CI: 1.18–2.50 and OR = 1.34, 95% CI: 1.14–1.58 respectively) living with their mothers and fathers.

Children with one delay were 3.9% versus 2.8% with multiple delays. The prevalence rate of the types of developmental delays of the surveyed children by numbers of delays is shown in Table 4, the prevalence of communication delay accounted for the largest number, with 2197 children (5.3%), followed by delay in daily life skills with 958 (2.3%), fine motor (FM) and socialization with (1.5% each). Gross motor (GM) was the least prevalent delay (1.3%).

**Table 4:** Prevalence rate of the types of developmental delays of the surveyed children by numbers of delays

Type of developmental delays (Surveyed children = 41,640)*	Children with a single developmental delay n = 1616 (3.9%)		Children with multiple developmental delays n = 1162 (2.8%)		Total DDs’ = 2778 (% of total surveyed)	
	no	Rate*/1000	no	rate*/1000	%*	rate*/1000
Gross Motor (GM) n = 543	130	3.1	413	9.9	(1.3%)	13
OR (CI) P multiple versus a single delay	6.30(5.08–7.82) ** 0.000					
Fine Motor (FM) n = 637	52	1.2	585	14.0	(1.5%)	15.2
OR (CI) P multiple versus a single delay	30.49 (22.61–41.13) ** 0.000					
Daily life skills n = 958	208	5.0	750	18.0	(2.3%)	23
OR (CI) P multiple versus a single delay	12.32(10.20–14.88) ** 0.000					
Communication n = 2197	1172	28.1	1024	24.6	(5.3%)	52.7
OR (CI) P multiple versus a single delay	2.81(2.28–3.46) ** 0.000					
Socialization n = 606	54	1.3	552	13.3	(1.5%)	14.6
OR (CI) P multiple versus a single delay	26.18(19.49–35.15) ** 0.000					
Total (rate/1000)	38.8		27.9		6.7%	66.7

* Percent and rate were calculated out of the total surveyed children (n = 41,640) per 1000, **highly significant < 0.01

The prevalence of multiple delays among children with developmental delays was slightly lower than that of a single domain delay. Each of the socialization and fine motor delays was most probable to be found as combined delays with other types of delays 26 and 30 times more than their presence as a single delay (OR = 26.18 and 30.49 respectively). Rates of different types of DDs are demonstrated in Table 4

When the distribution of developmental delay was studied year by year, it was found that children with at least one delay had the highest prevalence among children aged 7- < 8 years (8.6%) and children aged 5- < 6 years (8.6%) followed by children aged 3- < 4 years (8.3%).

The highest prevalence of gross motor, daily life skills delay, and socialization delay was found among children aged 3- < 4 years (2.9%, 4.1%, and 2.3% respectively). The highest prevalence of fine motor delay was found among children aged 5- < 6 years (3.7%). The prevalence of communication delay was highest for children aged 7- < 8 years (7.9%). The data regarding the distribution of developmental delays’ domains by age is demonstrated in Table 5

**Table 5:** Distribution of Developmental delays’ domains by Age out of 41,640 surveyed children

Type of developmental delays		Age group													
		12–< 15 ms	15–< 18 ms	18–< 24 ms	2–< 3 years	3–< 4 years	4–< 5 years	5–< 6 years	6–< 7 years	7–< 8 years	8–< 9 years	9–< 10 years	10–< 11 years	11–12 years	Total
Total no surveyed		1304	968	1941	4170	4205	4349	4379	3792	3632	3450	3204	3239	3007	41,640
Gross Motor	Sum	0	11	52	68	124	52	105	61	70	0	0	0	0	543
	Column %	0	1.1%	2.7%	1.6%	2.9%	1.2%	2.4%	1.6%	1.9%	0%	0%	0%	0%	1.3%
Fine Motor	Sum	14	0	14	42	97	108	160	99	103	0	0	0	0	637
	Column %	1.1%	0%	0.7%	1.0%	2.3%	2.5%	3.7%	2.6%	2.8%	0%	0%	0%	0%	1.5%
Daily life skills	Sum	0	0	18	42	173	155	166	124	103	69	65	0	43	958
	Column %	0%	0%	0.9%	1.0%	4.1%	3.6%	3.8%	3.3%	2.8%	2%	2%	0%	1.4%	2.3%
Communication	Sum	0	10	64	191	245	129	265	246	288	237	188	174	159	2196
	Column %	0%	1.0%	3.3%	4.6%	5.8%	3.0%	6.1%	6.5%	7.9%	6.9%	5.9%	5.4%	5.3%	5.3%
Socialization	Sum	13	7	24	30	98	53	56	46	62	48	47	80	42	606
	Row %	1.0%	0.7%	1.2%	0.7%	2.3%	1.2%	1.3%	1.2%	1.7%	1.4%	1.5%	2.5%	1.4%	1.5%
Children with at least one DD	Sum	20	20	81	228	351	290	375	290	314	257	202	181	169	2778
	Column %	1.5%	2.1%	4.2%	5.5%	8.3%	6.7%	8.6%	7.6%	8.6%	7.4%	6.3%	5.6%	5.6%	6.7%

**Fig. 1:**
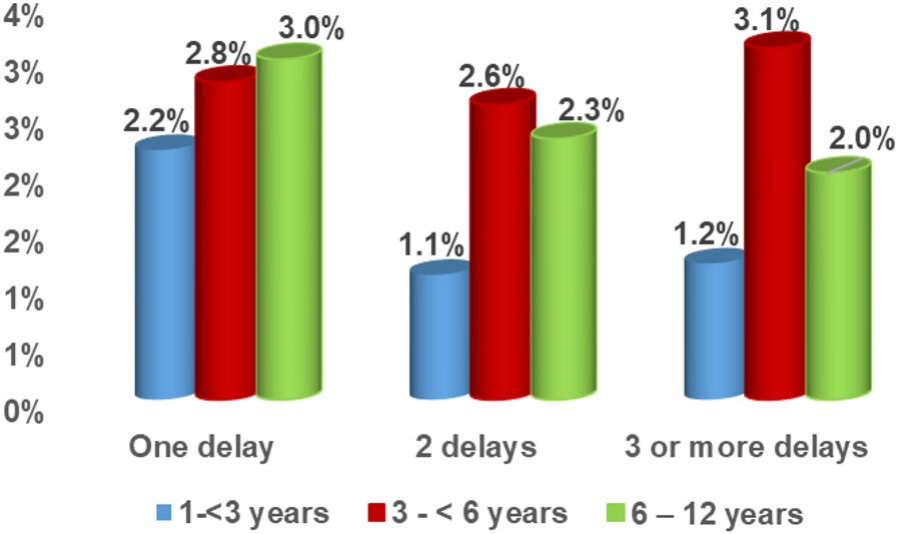
Profile of developmental delays by the age category of the surveyed children

The highest prevalence of one delay was among children in the age range 6–12 years (3%). Whereas the highest prevalence of two delays and multiple delays was among children in the age range 3– < 6 years (2.6%, 3.1% respectively) (Fig. 1).

Table 6 shows the data of the multivariate logistic regression model for predictors of developmental delays, 19 variables (11 sociodemographic/epidemiological, two maternal risk factors, and six children risk) were entered into a multivariate logistic regression using the enter selection procedure to explore the predictors of single developmental delay and the multiple developmental delays. The final model had a good fit and contained 8 variables increasing the association for developmental delays (2 sociodemographic/epidemiological, 1 maternal risk factor, and five children risk factors). Risk factors in order were: Children suffering from any convulsions increased odds of DDs by more than three times compared to healthy children (OR = 3.09; 95% CI: 2.47–3.86), Birth weight less than 2.5 kg (OR = 1.94; 95%, CI: 1.66–2.26), Mothers having any health problem during pregnancy increased the odds to have DDs by more than one and half times compared to healthy mothers and normal birth weight children (OR = 1.70; 95%, CI:1.48–1.95). Being a boy child in the middle social class or suffering from cyanosis or kept in an incubator for more than two days increase the odds to have DDs by almost one and half times compared to healthy children (OR = 1.75; 95%, CI: 1.61–1.9, OR = 1.41, 95%, CI: 1.29–1.53, OR = 1.65, 95%, CI: 1.26–2.14 & OR = 1.46, 95%, CI: 1.27–1.67 respectively).

**Table 6:** Multivariate Logistic regression model for predictor of developmental delays (With total delays vs without delays)

Parameters	With total delays vs without delays			One delay vs multiple delays		
	Wald	OR	CI	Wald	OR	CI
Age	9.157	1.02**	1.01–1.03	14.35	0.95*	0.91–0.07
Sex	166.9	1.75**	1.61–1.9	0.180	1.04	0.88–1.23
Locality (urban–rural) urban is the base	5.66	1.13*	1.02–1.26	3.81	1.23	0.99–1.51
Social level (high–middle–low) low and high are the base						
Middle to (low and high)	60.11	1.41*	1.29–1.53	0.21	1.04	0.88–1.24
Location (lower–upper–frontiers) is the base						
Lower to cities	41.02	0.65**	0.57 -0.74	4.09	1.31*	1.01–1.70
Upper to cities	97.72	0.5**	0.44-0.58	22.16	1.99**	1.12–1.88
Frontiers to cities	84.83	0.45**	0.38-0.53	14.56	1.93**	1.38–2.71
Maternal age at birth	1.49	1.0	0.98–1.0	5.11	1.02	0.99–1.03
Maternal University and above to less	39.02	0.58**	0.49-0.69	1.13	0.84	0.60–1.16
Paternal University and above to less	39.59	0.60**	0.54-0.76	0.75	0.87	0.64–1.19
Maternal work status (work–unemployed and does not look for work) base is working	0.02	1.0	0.88–1.14	2.39	0.82	0.63–1.06
Mothers have any health problems during pregnancy	55.29	1.70**	1.48–1.95	1.05	0.87	0.68–1.13
Difficult labor	1.35	1.07	0.95–1.21	14.81	1.55**	1.24–1.94
Child born less than 7 months (preterm pregnancy)	0.97	0.88	0.82-0.1.0	0.001	1.0	0.56–1.76
Baby’s weight less than 2.5 kg at birth	70.16	1.94**	1.66–2.26	0.14	1.06	0.80–1.39
Children suffer from jaundice after birth	7.99	1.15*	1.04–1.26	2.70	0.86	0.71–1.03
Children suffer from bluish discoloration after birth (cyanosis)	13.74	1.65**	1.26–2.14	6.20**	1.69**	1.12–2.56
Children suffer from any convulsions	98.63	3.09**	2.47–3.86	4.45	1.46**	1.12–2.56
Children kept in an incubator for more than two days	29.71	1.46**	1.27–1.67	2.66	1.23	0.96–1.57
Constant	330.1	0.044		6.56	0.43	

*Significant, **highly significant

Five sociodemographic/epidemiological factors were found to be protective against the occurrence of DDs. The protective factors that decrease the odds to have DDs were being resident in Frontiers, upper or Lower Egypt, decrease the odds to have DDs by 55% 50% and 35% compared to being living in cities (OR = 0.45, 95%, CI: 0.38–0.53, OR = 0.50, 95%, &CI: 0.44–0.58 and OR = 0.65, 95%, CI: 0.57–0.74). Paternal and maternal education with a university degree or above degree decreases the odds to have DDs by 40% compared of being with less degree of education (OR = 0.58, 95%, CI: 0.49–0.69, OR = 0.64, 95%, CI: 0.54–0.76).

Out of all the studied risk factors, six were found to be associated factors for multiple DDs. Children living in upper or lower Egypt or frontiers carried higher than one and a half higher odds than other localities for multiple DDs with varied significant levels. Mothers who experienced difficult labor or who had children who suffered from cyanosis or convulsions after birth was one and a half more likely to have multiple DDs than healthy newborn.

## Discussion

This study provides a national estimate for the prevalence of developmental delays among 41,640 Egyptian children, aged from 1 year up to 12 years, using a reliable screening tool. The overall prevalence of children with DDs was 6.7%. The detected overall prevalence of DDs is within the estimated range of prevalence of DDs for the pediatric population, which varied between 5 and 15% [5, 30, 31]. This prevalence varied among different stages of child development. Preschool children aged 3– < 6 years had the highest prevalence of any DD 7.9%, followed by the school-age group (7.0%) and the least prevalence was among infants and toddlers (4.2%). The distribution of delay in each year of age presented a different prevalence. The highest prevalence of DDs was among children aged 7– < 8 years (8.6%) and children aged 5– < 6 years (8.6%), followed by children aged 3– < 4 years (8.3%). Whereas, in a global study done by Gil and his colleagues [19], which included 63 low and middle-income countries, a very wide variation in estimated developmental delays was reported across countries, from nearly 70% in Chad to 3% in Barbados. Previous literature assumed that, in low-income countries, precise statistics cannot be obtained because of the difficulty in adopting culturally and linguistically screening tools, used to detect development delay [32]. Meanwhile, some children with suspected delay can have a developmental disability, it is worth mentioning that this is not the basic rule, as the presence of a delay does not necessitate the presence of a disability [19].

Overall, the present study showed that the most prevalent type was Communication delay (5.3%) which denoted cognitive and/ or language delay. Cognitive delay is one of the most prevalent types of DDs as supported by previous studies [14, 33]. Impaired cognitive functioning as demonstrated by delayed language acquisition, literacy, numeracy, and independence may continue across childhood and is likely to be accompanied by intellectual disability with its deleterious lifelong impact on health and well-being [33]. Lower prevalence was identified for gross motor delay (1.0%), fine motor delay, and socialization delay (1.5% each). These estimates are in close agreement with the results of a cross-sectional study in Egypt [7]. On the other side, in contrast to our findings regarding the prevalence of motor delays, higher rates were reported by Villa and his colleagues [4]. Nevertheless, motor development has been assumed to differ in prevalence in children from different cultural backgrounds [34]. According to the distribution of different types of DDs per age group, the current study revealed that concerns about certain developmental problems are highly age-related. For example, children aged 7–< 8 had the highest prevalence of communication delay (7.9%). This may be related to environmental neglect or abuse, could be a sign of a learning problem that may not be diagnosed until the school years or may be a sign of intellectual disability [35]. Besides, the increased parental concern about delayed school performance in this age. While the highest prevalence of fine motor delay (3.4%) was in children aged 5—< 6, which may also be associated with entering school when there is an increased need for academic skills such as the use of pencils. This also may be attributed to coexisting attention deficit hyperactivity disorder (ADHD) at this age group. Children with ADHD often display deficits in tasks requiring coordination of complex movements, such as handwriting [36].

Generally speaking, there are difficulties in comparing estimated prevalence rates either of any type of developmental delay or a particular type of DDs due to a number of methodological concerns, such as using different tools for developmental assessment. Other factors may be related to the type and number of the affected domains [37] or to the type of studied population. Likewise, this study was conducted in community-based settings, contrary to some studies with higher prevalence rates that were conducted on high-risk populations [38].

The highest prevalence of one delay was among children in the age range 6–12 years. Whereas the highest prevalence of two and more delays was among children in the age range 3– < 6 years. Meanwhile, the link between the number of delays experienced by children and their lifetime prevalence has not been studied before. Moreover, it has been reported that DD in one domain is often found to be combined with a delay in other domains [39]. In the current study, the risk of having multiple delays was significantly higher in comparison to isolated delays in all the studied domains. Otherwise, the most probable combined delays to be found were socialization and fine motor domains.

It is well acknowledged that various risk factors are involved in the etiology of developmental impairments, including biological, social, and environmental [13, 40]; and so recognizing crucial associated factors for child development will provide the chance to establish favorable environmental settings and allow early intervention for optimum development [4]. Regarding the epidemiological characteristics, we found that age emerges as a determinant factor; children aged 3– < 6 years have the highest prevalence, with almost twice the odds that of the age group 1– < 3 years but slightly more than children aged 6–12 years. This could be attributed to the fact that at the age of 3 to 6 years most of the children have been engaged with other children either for the nursery or at the beginning of formal education so any slowness of milestones could be easily detected. Broadly, that was close to the findings in previous literature [5, 31]. In addition, this age group are the most vulnerable to be subjected to malnutrition, under stimulation, maltreatment and frequent trauma [2].

On the other side, boys were one and three quarters more likely than girls to be diagnosed with any developmental delays. The gender differences reported in the current study, have also been proved in previous studies [4, 31, 41]. Possible explanations may be that boys exhibit more externalizing behaviors, develop some skills later than girls [42].

Furthermore, the current study found that the geographical and sociodemographic distribution have a profound influence on the probability of the occurrence of DDs. The study revealed that children belonging to urban localities have a higher odd of DDs than rural residents. The association between urban/rural residence and the prevalence of DDs is inconsistent [5, 43]. Both rural and urban people have particular problems, that may influence childhood development. For example, in urban areas, overcrowding, and environmental pollution. On the opposite side, rural residents may face problems such as a shortage of infrastructure and lower socioeconomic levels. On the other hand, robust family bonds and social relations in rural communities, are likely considered protective factors. Existing evidence proposes that protective factors can alleviate the risk and contribute to optimistic development [44]. Another reported risk factor for multiple DDs is living in upper or lower Egypt or frontiers-which was on contrary to that of single DD- it carried almost one and half higher odds than living in cities. This may be explained by differences in socio-demographic characteristics and risk factors in these regions, including higher financial constraints, and lack of basic resources. Furthermore, belonging to the middle socioeconomic class was found to be a risk factor for single or multiple DDs. Surprisingly, the association was the same for children belonging to both the low and high classes. It is unclear what mediates the influence of socioeconomic status on child development whether parental education, availability of resources or parenting style. Lue et al. [45], found no link between parenting styles and poverty. On the other hand, Correia et al., [31] reported a positive association between poverty and DDs. Other authors claimed that a lack of supportive parenting may impact children even raised in rich families [46]. It can be assumed that knowledge is more essential than wealth, as it represents the keystone for optimal parenting. Another research in Egypt supports our findings, demonstrated that middle social class parents showed inadequate knowledge about their children’s development. [47].

By analyzing the impact of parental characteristics, we found that living without mothers and/or fathers in homes was associated with increased odds of having DDs by one and half times. This finding supports the earlier literature that affirmed a significant association between family structure and a child’s intellectual and emotional development [48]. Hence, it has been reported that having a complete family is a good indicator of proper parent–child interaction [49], which in turn stimulates early developmental outcomes. On the other hand, in accordance with our findings, parental education was defined as the main factor in child development [43, 50]. Higher parental education was a strong protective factor in our results; it decreases the odds to have DDs by 40% compared to being with less degree of education. Again, higher parental education enhances the quality of caregiver-child interaction [45].

On looking up for DDs predictors, prenatal, natal, and postnatal risk factors showed a significant impact. As we found that, neonatal convulsions and, low birth weight strongly predict DDs. Moreover, other perinatal factors such as maternal health problems, neonatal cyanosis, and newborn kept in an incubator for more than two days, potentiate the risk of DDs by almost one and half times. Furthermore, difficult labor and neonatal cyanosis were strong predictors for multiple DDs. Accordingly, the influence of perinatal risk factors has been reported before in literature. Neonatal convulsions as a contributing factor for DDs were reported by Chattopadhyay et al. [51]. While, Sharma et al. [14], found a significant association between neonatal illnesses, low birth weight, and delayed development. The possible reason for this association is the occurrence of placental dysfunction, birth asphyxia, and neonatal hypoglycemia which all, predict developmental impairments [14, 52, 53, 53]. In Egypt, El Meliegy and El Sabbagh [55], found high rates of perinatal risk factors and neonatal complications in association with DDs, which indicates insufficient maternal and child health care. A good point is that the majority of influencing factors are preventable and could be changed by either health education for the environmental factors [54, 55], and improving caregivers’ behaviors [56, 57], or by nutrition supplements and school snacks for school children [58] as proved by many studies in Egypt. In addition, a randomized interventional Egyptian study for enhancing women’s rights for having their governmental perinatal care at the highest quality proved reduction of a lot of mothers and child risk factors leading to DDs [59]. Furthermore, improving women’s care-seeking behaviors during the perinatal period positively impacted and improved birth outcomes [60].

## Conclusion and recommendation

The detected prevalence of DDs is within the estimated range of prevalence of DDs for the pediatric population. The majority of the detected risk factors are preventable. Developmental screening is recommended to be implemented in all primary care settings as a routine practice, to promote early detection and intervention in suspected cases of delayed development. This study highlighted the risk factors that increased the association of having any types of developmental delays and who should be monitored at maternal and child health care facilities including children suffering from any of the following after birth; convulsions, cyanosis or kept in an incubator for more than two days. Also, the baby’s weight of less than 2.5 kg at birth, mothers having any health problems during pregnancy, or having a baby boy was associated with all kinds of DDs. Moreover, mothers who experience difficult labor or who have children suffering from cyanosis after birth were at increased risk for having multiple DDs.

The outcomes of this study can contribute to the body of evidence that supports and eventually enhance community education, developmental screening, and diagnostic efforts to improve early identification and proper management for DDs in children.

## Strengths of the study

This national survey highlighted the most important contribution to the need for community-driven data to detect the preventable types of disabilities that are dependent on developmental delays. DDs when neglected can lead to serious disabilities that could impact not only the life of the children but the whole family. This study was the first to highlight not only the national prevalence of developmental delays and their risk factors, but the study focused also on the prevalence of multiple developmental delays and their risk factors through using reliable and highly sensitive tool which is considered as being diagnostic tool rather than only screening one and verified by specialists. Accordingly, the provided estimates could be considered as accurate as well as reliable. The study also provided the protective factors against having DDs. In addition, this study was the first to be done at a community-based level that targeted a very wide and large sector of children’s population aged 1 to 12 years amounted to 41,640 children at the end stage.

### Study limitations

This study has some limitations. Environmental factors that were suggested by this study to be high-risk factors behind the high probability of having developmental delays among urban communities and cities were not investigated in the study. The influence of the nutritional factors although well documented to affect development in many Egyptian studies both early in life due to exclusive breastfeeding [61], and proper weaning and later on during childhood [60, 62], yet it could not be studied because of the nature of this study which was a cross-sectional screening questionnaire that should be administered in 15–20 min or less. A thorough investigation of the nutritional patterns is known to take a long time.

## Supplementary Information

**Additional file 1.** Raw data.

## Data Availability

All data generated or analyzed during this study are included in this published article [and its Additional file 1].

## Abbreviations

CAPMS: The Central Agency for Public Mobilization and Statistics
CDC: Cairo Demographic Center
CI: Confidence interval
DDs: Developmental delays
FM: Fine motor
GM: Gross motor
MOHP: The Ministry of Health and Population
OR: Odds ratio
VABS: Vineland Adaptive Behavior Scales
